# Adapting Master’s-Level Chemistry Education in the United States: Insights from Industry, Academia, and the Literature

**DOI:** 10.1021/acs.jchemed.5c00418

**Published:** 2025-12-12

**Authors:** Brian Johnson, Amanda Lindell, Nina M. Goodey

**Affiliations:** † 688890 Inform Evaluation & Research, Amherst, Massachusetts 01002, United States; ‡ Department of Chemistry and Biochemistry, 8087 Montclair State University, Montclair, New Jersey 07043, United States

**Keywords:** Master’s-level chemistry education, experiential learning, professional skill development, workforce readiness, course-based research experiences, curriculum innovation

## Abstract

As the chemical industry evolves in response to technological and global challenges, Master’s-level chemistry programs must adapt to prepare graduates for workforce success. This article explores how such programs can better integrate professional skills such as communication, collaboration, and research. Drawing on literature and stakeholder interviews from a review of the Chemistry M.S. and Pharmaceutical Biochemistry M.S. programs at Montclair State University, a Hispanic Serving Institution in New Jersey’s pharmaceutical hub, we identified four key themes: the importance of hands-on research experiences, the integration of professional skills development, the need for flexible course and credit requirements, and the challenges of sustaining small programs. While independent research offers valuable experience, access can be limited by students’ financial and time constraints. Financial support and even brief, course-based research opportunities can mitigate this gap. Using experiential learning and communities of practice as guiding frameworks, we offer actionable recommendations for increasing curriculum relevance, equity, and workforce alignment. The insights are broadly applicable to institutions seeking to modernize master’s level chemistry education for a diverse student population.

## Graphical abstract

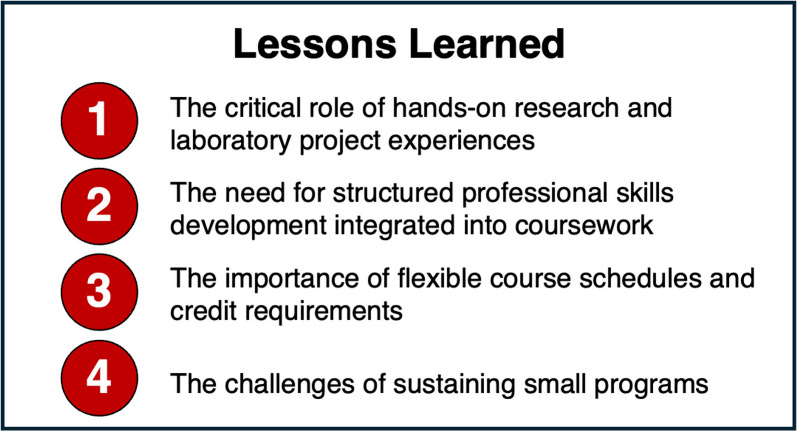

## 1 Introduction

With the rapid evolution of technology and the pressures of global issues such as climate change, chemical industries are adapting to meet the needs of a changing planet and human population. Master’s degree programs in chemistry must similarly evolve to continue meeting the needs of industry and society. But what is the role of a master’s program in preparing chemists in professional skills such as communication, collaboration, research, and networking? Should chemistry and biochemistry master’s programs “stick to the science” and leave professional preparation to other aspects of the student’s training and preparation for the workplace? With funding from the National Science Foundation (NSF), Montclair State University is in the midst of a five-year project to evaluate and reimagine its Chemistry M.S. program. This process includes consulting relevant literature and interviewing chemical industry professionals in the Northeast, Montclair chemistry faculty, and Montclair chemistry master’s students regarding expectations of Chemistry M.S. graduates. In this article, we reflect on key themes that have emerged through this process, including identifying “lessons learned” for other institutions seeking to position their Chemistry M.S. programs for the future. We pay particular attention to how best to integrate research experiences and professional skills into a master’s program of study.

To critically analyze the emerging trends, this article applies experiential learning theory to reflect on how hands-on experiences contribute to conceptual understanding and skill development.^1^ Experiential learning theory emphasizes learning through experience and involves a cyclical process of experience, observation, conceptualization and experimentation, elements that are relevant to hands-on learning experiences in master’s level education. Additionally, communities of practice and situated learning theories are utilized to discuss how industry collaborations, professional networks, and skill development can influence students’ transition from academia to workforce.^2–4^ These frameworks provide a structured approach to discuss how students gain expertise through participation in research groups and the effects of professional skills integration within coursework and research experiences on student outcomes. In this article, we use the term “professional skills” to refer to skills not specific to chemistry, but which are important in chemistry workplaces. We acknowledge that in some cases, there may overlap between “professional skills” and “technical” chemistry skills. For example, learning to use a spreadsheet could be considered a professional skill, but it could also be viewed as a technical skill in the context of lab data analysis.

## 2 Background and Context

Graduate students often face financial pressures and family obligations, which can make it difficult to complete their programs. This can be especially true for first-generation or low-income students balancing work, commuting, and cultural expectations with their education.^5^ Surveys show that about 30% of graduate students have children, and many also provide care for other relatives.^6,7^ Women in STEM fields face additional pressures from gendered expectations, which can intensify stress and impact academic performance.^8,9^

Montclair State University, a designated Hispanic-Serving Institution (HSI), primarily serves a low-income population. Approximately 52% of first-year, full-time undergraduate students receive Pell grants, compared with the national rate of 32%.^10^ Approximately 44% of Montclair students are first-generation college students. The Chemistry and Biochemistry Department M.S. programs serve a diverse student body that differs from national averages, suggesting a need for tailored adaptations, including program flexibility and diverse faculty and staff representation. From 2017 to 2025, the programs’ self-reported racial and ethnic composition averaged 9% Asian, 23% Black/African American, 20% Hispanic, 42% White, and 6% Multiracial. National data show that master’s degree recipients in physical sciences are 5% Black/African American and 8%, highlighting Montclair’s higher representation of underrepresented groups.^11^ Similarly, a 2017 Council of Graduate Schools survey reported Hispanic graduate students comprised 10% in chemistry.^12^ Gender distribution in the Montclair programs is 58% female and 42% male, compared with 44% female nationally.^11^ Montclair also serves a substantial but smaller proportion of temporary resident students (30%) than the national average (35%).

Within these programs at Montclair, graduate students anecdotally report extensive family obligations, needing to work because of the high cost of living in Northern New Jersey, and long commutes. These external considerations can hinder progress and push some to pursue part-time study. To better serve its population, the department has revised entry and graduation requirements, updated the core curriculum, and challenged traditional expectations of the education of professional chemists and biochemists. In the following sections, we share relevant literature, four key themes from this process, and lessons that may apply to other institutions.

Chemists with a master’s degree in the United States have favorable career prospects. According to the American Chemical Society, chemists with an M.S. degree reported a median salary of $98,000.^13^ The unemployment rate for chemists in 2022 was 0.6%, well below the national rate. The U.S. Bureau of Labor Statistics (2018) notes that while a Bachelor’s degree suffices for entry-level chemist roles, a master’s degree enables advanced roles in research and development.^14^ Employment of chemists and biochemists is projected to grow 8% and 9%, respectively, from 2023 to 2033, faster than the average for all occupations. For students seeking more than entry-level laboratory positions, a master’s degree can provide higher starting salaries and access to research roles, especially in pharmaceuticals and biotechnology. Given Montclair’s location in the New Jersey pharmaceutical hub, ensuring that its programs prepare students for these roles is especially important.

## 3 Methods

Over the past two years, Montclair State has carried out evaluation to identify changes that would better serve its Chemistry M.S. students. External evaluators from Inform Evaluation & Research gathered data through interviews with Chemistry industry professionals (n = 11), Montclair Chemistry students and alumni (n = 20), and Montclair Chemistry faculty (n = 15). We interviewed only tenured or tenure-track faculty for this project because adjunct faculty only occasionally teach graduate courses in our department. As such, the views of adjunct faculty are not represented in our findings. Interviews with industry professionals included questions on skills and competencies to prioritize in M.S. programs, what they look for when hiring recent M.S. graduates, and the role of graduate education in fostering nontechnical skills. Interviews with students and alumni focused on the value of hands-on experiences and nontechnical skill development in their M.S. coursework, the impact of recent changes to Montclair’s Chemistry M.S. program, and obstacles they faced in completing an M.S. degree. Interviews with faculty focused on their perceptions of changes to the M.S. program, and the role of graduate education in fostering nontechnical skills. Interviews ranged in length from 20 to 30 min, and were recorded and transcribed. Analysis was carried out using open coding to identify emergent themes and any outlier insights. This study was approved by the Montclair State University Institutional Review Board (IRB approval number: IRB-FY19–20–1866). No unexpected or unusually high safety hazards were encountered.

## 4 Insights from the Literature

We reviewed literature relevant to Montclair State’s internal assessment of its Chemistry M.S. program. This scan was not an exhaustive literature review. However, we carried out keyword searches in Google Scholar and Google. “Graduate Chemistry Education” was used exclusively in Google Scholar and produced a large number of articles; we therefore focused our scan on more recent literature. Search terms such as “trends in chemistry”, “future of chemistry”, “New Jersey chemistry industry”, “pharmaceutical industry trends”, and “chemistry industry trends” were more fruitful in general Google searches to find industry white papers. A summary is presented below of three key areas in the literature: (1) trends in graduate chemistry education; (2) trends in chemical industries; and (3) the future of chemistry.

### 4.1 Trends in Graduate Chemistry Education

Hands-on research projects are essential in master’s level chemistry education, supporting concrete experiences and active experimentation, which are key stages of Kolb’s experiential learning cycle.^15^ These experiences enable students to apply theoretical knowledge to real-world applications, building understanding and practical skills. Kondo and Fair examined the skills gap in undergraduate chemistry education and found that while employers expect and value technical proficiency in chemistry, they see gaps in leadership skills such as teamwork, communication, and interpersonal skills.^16^ They noted that these skills can be developed through research experiences and internships, while general leadership training is not effective. For example, teamwork skills should be honed in the context of a technical chemistry project. Moreover, managers in the chemical industry rank the following skills as most important out of 52 skills: understanding of concept behind methods, procedures, assays one works with, possessing problem-solving skills, and an ability to learn new methods.^17^ These skills are best learned through hands-on research projects.^18^ Incorporating research-projects, team-based projects, and communication training into coursework can better align graduate education with industry expectations and improve employability.

In addition to content knowledge, chemistry graduate students need professional skills such as communication, collaboration, problem-solving, professionalism, and networking regardless of their career sector.^19,20^ Professional skill development and professional networking provide students with opportunities for legitimate participation in communities of practice, fostering professional identity development and easing transition to the workforce. As noted by Kondo and Fair, development of professional skills takes place optimally in the context of research and internship experiences.^16,21,22^ Developing competencies in effective communication, negotiation, leadership, networking, interpersonal skills, and active listening is essential for navigating diverse STEM environments. These six skills have been successfully implemented in undergraduate research settings to enhance student persistence and success, particularly for marginalized students, and can similarly be applied at the master’s level to support career readiness and professional growth.^23^

The literature scan also suggested that teaching critical thinking skills such as problem-solving and systems thinking across the master’s curriculum will help students when they encounter new challenges in a rapidly changing world.^19^

For example, green chemistry approaches across a curriculum will not only help reduce the environmental impacts of chemistry education and research, but also prepare students for growing calls for sustainability around the world.^24^ In China, for example, chemistry master’s programs have shifted toward interdisciplinary education that aligns with national policy objectives, increasingly emphasizing problem solving, creativity, sustainability and green chemistry.^25^ This also aligns with the continued trend in interdisciplinary studies in chemistry as systems thinking strives to make connections between parts of complex systems that are not bound by traditional disciplinary lines.^26^

Finally, our literature scan suggested that, similar to helping students develop career-enhancing “soft skills”, graduate programs in chemistry must be seen as welcoming and supportive environments with relevant policies and procedures, and diverse faculty and staff. Strategies to support students include training in effective time management,^27^ institutional resources such as flexible scheduling, childcare, and peer mentoring,^28,29^ and access to mental health services.^30^ Universities offering these supports can help chemistry graduate students manage financial needs and the demands of family and academic commitments. Finally, increasing calls for flexibility in programs of study need to be accompanied by programs and policies that help faculty adjust to rapidly shifting demands and avoid burnout.^31,32^

### 4.2 Trends in Chemical Industries

Chemical manufacturing and biopharmaceuticals are important industries in New Jersey. In the near-term, chemical manufacturing will be important in key industries such as construction and the automotive industry as they continue to rebound after the COVID-19 pandemic.^33^ Sustainability continues to be an important trend and opportunity for the chemistry industry in both the near- and long-term. New regulations and consumer pressure should drive the industry to clean up its processes and reduce carbon emissions while also providing opportunities for innovation to accomplish those goals in other industries and contribute to overall solutions to environmental problems.^33,34^ These pressures are reflected in the pharmaceutical industry as well. Personalized medicine will be an important market force moving forward in the research and development of this sector.^35^ These forces create challenges and opportunities for chemical industries and should be considered in strategic planning.

### 4.3 The Future of Chemistry

When looking even longer-term at chemistry, authors have looked at how larger global trends and forces will impact industry, education, research, and manufacturing.^36^ Climate change impacts and mitigation will shape what chemistry focuses on and how it works. Problem-driven research related to health and public health crises, energy, water, and food systems will become increasingly popular and will require more interdisciplinary work. The Royal Society specifically forecasts (1) decreased funding for basic research, (2) increased need for science communication, (3) greater need for interdisciplinary work, and (4) strong leadership to shape society’s view of chemistry.^36^

## 5 Evaluation Results

### 5.1 Theme #1: The Importance of Hands-on Learning

In our evaluation, chemistry faculty, students, and industry professionals all agreed that hands-on research and lab-based experiences are essential components of a Chemistry M.S. degree. Students valued internship and research thesis opportunities, in particular. As one student shared, “[Hands-on experience] is very important because most of the students here are trying to go into a really tough job market, so more hands-on experience with different lab techniques would definitely help.”

Faculty also saw the value of hands-on learning, though opinions on how much hands-on content should be included in courses varied. “I think that a practical component seems very appropriate and important for a master’s program,” said a faculty member. “And maybe also something that makes us unique.”

Lab-based activities are the most common hands-on approaches at Montclair State, with some faculty not able to identify other hands-on options that would be relevant to chemistry courses. “Other than having the lab component, I’m not really sure about other hands-on kind of activities at the master’s level that would be appropriate”, one faculty member commented. Table 1 summarizes examples of how Montclair Chemistry faculty have increasingly incorporated hands-on learning into M.S. courses and some of these can be seen as graduate level CUREs.^37^ However, some faculty expressed that not all courses need a hands-on component and noted that lab time for evening courses is difficult to coordinate.

**Table 1:** Montclair State Chemistry and Biochemistry Department hands-on grad course experiences

CHEM560 Advanced Analytical Chemistry	This course dedicated 2 weeks for hands-on lab experience. The emphasis of the course was to develop an understanding of how uncertainties in measurements depend on quantity and quality of the data, how uncertainties propagate through calculations, and how instrumentation and protocols affect uncertainties. This knowledge was applied to analyze data that students generated in the laboratory. The lab focused on ion concentration determination where students used three independent instrumental methods of titration, atomic spectroscopy, and absorbance (after a chemical reaction) to compare the errors and conclude their final values of the ion concentration. The students were responsible for full data analysis and producing a report from the laboratory experience, which aimed to help them make connections between the statistical techniques and the actual data analysis.
CHEM582 Biochemical Pharmacology	Students engaged in practical lab activities that intended to enhance their understanding of drug–protein interactions. They purified enzymes, measured protein concentrations, incubated enzymes with different drug molecules, and set up protein crystallization experiments in sitting-drop format. They examined wells for crystal growth using microscopy. Additionally, they used Chimera software to visualize protein-drug interactions in complexes and participated in an interactive session on protein structure determination. A highlight of the course was a visit to Brookhaven National Laboratory (BNL), where students had the opportunity to see protein structure determination via X-ray in real life.
CHEM590 Interfacial Science & Engineering: Polymer Synthesis & Self-assembly	Students gained hands-on experience with polymer synthesis techniques, including step-growth, ring-opening and controlled free radical polymerization, and explored polymer characterization methods such as proton NMR, gel permeation chromatography (GPC), thermogravimetry analysis (TGA), differential scanning calorimetry (DSC), and transmission electron microscopy (TEM). They engaged in air-free synthesis and microwave-assisted polymer preparation, learning skills beyond traditional Organic Chemistry laboratories. Over 11 lab activities, students synthesized, purified, and characterized polymers, critically analyzing data from various analytical instruments. The course’s interdisciplinary approach aimed to deepen their understanding of polymer chemistry and spark enthusiasm for research.
CHEM536 Nuclear Magnetic Resonance (NMR): Theory and Practice	Students gained hands-on experience with both the theory and practical applications of NMR in analyzing small organic molecules and polymers. They engaged in lab sessions that included proton and carbon NMR spectral interpretation, polymer end-group analysis, 2D NMR spectral interpretation (e.g., COSY, HSQC, etc.) and mastering the MNOVA software. Students also engaged in interactive discussions, where they collaboratively analyzed spectra, and learned from guest lectures that connected NMR techniques to cutting-edge research in protein structure and Li-ion battery characterization. This immersive approach aimed to help students build confidence in using NMR as a powerful tool in organic chemistry.
CHEM590 Special topics in Chemistry: Computational Chemistry	One meeting per week was used to study the methodology of the field, and one meeting per week was reserved for hands-on projects. Students used a modern quantum chemistry program to model the electronic structure of a variety of molecules. Examples of calculated properties include vibrational frequencies, IR spectra, NMR spectra, interaction energies, barrier heights of chemical reactions, and free energies of reaction. The course aimed to provide students with a hands-on experience of computational chemistry. Intended student outcomes included an understanding of the strengths and limits of commonly used methods, and a deeper understanding of the theoretical framework behind the methods.
CHEM579 Biomolecular Assay Development	Students gained hands-on experience with the drug discovery process by establishing enzymatic and molecular screens to identify small molecule inhibitors targeting therapeutically relevant enzymes. After lectures on biomolecular screening techniques and drug discovery workflows, students applied this knowledge in the lab, developing their own screening assays. One project involved creating a beta-lactamase assay to identify novel inhibitors. Students expressed and characterized recombinant beta-lactamase, assessed enzyme kinetics and assay conditions, and calculated Z-factors to evaluate assay robustness. They then determined IC_50_ values for known inhibitors and screened a diverse small molecule library from sources such as the NCI and MMV. The intended student outcomes for this project were to gain a comprehensive understanding of the practical steps involved in early stage drug screening in an industrial context.

Industry representatives also recommended prioritizing hands-on research or lab experiences in Chemistry M.S. programs. They noted that this experience gives graduates a competitive edge. “We tend to prefer master’s chemists who have done research in their education,” said an industry representative. “If someone has a purely class-based master’s...without the research component, it’s really unclear if they have the skills necessary to work in a laboratory.”

These findings indicate that experiential learning activities, including research projects, are viewed as important for improving students’ problem-solving skills and conceptual understanding. According to Kolb’s experiential learning theory, these experiences provide opportunities for concrete experiences, reflective observation, and experimentation.

To respond to program evaluation findings, several courses M.S. courses at Montclair have incorporated new or expanded hands-on components. For example, CHEM560 (Advanced Analytical Chemistry) added a two-week laboratory module in which students generated their own data to analyze uncertainty, strengthening their understanding of error propagation. CHEM582 (Biochemical Pharmacology) implemented a protein crystallization project, paired with a Brookhaven National Laboratory site visit, to reinforce structural biology concepts in drug discovery. CHEM590 (Polymer Synthesis & Self-Assembly) expanded to include advanced polymer characterization methods, allowing students to connect synthesis with real-world analysis techniques. These changes address industry feedback, emphasizing the need for graduates to demonstrate laboratory experimental design, as well as data analysis skills and familiarity with modern laboratory techniques.

A department interested in making similar changes can conduct a curriculum audit to identify courses that can be revised to incorporate a lab or research project. Criteria that departments may use to prioritize courses include (1) opportunities for integrating lab or research-based projects that reinforce core concepts or foster workforce-relevant skills, (2) feasibility of implementation given the course format (e.g., enrollment size), (3) faculty expertise and interests, and (4) resource and instrumentation availability. While expanding hands-on experiential learning is valuable, project-based and CURE-style laboratories require more resources. These higher costs include specialized equipment and consumables. Phased implementation where initially only one experiment/mini project is introduced into a course can help address financial challenges. Moreover, hands-on lab modules can be mixed with computational or data analysis modules to conserve resources. Finally, identifying external funding opportunities through grants, foundations, or industry partnerships may be practical for some departments.

*Lessons Learned*

- *Chemistry M.S. programs should identify courses that would benefit from a lab or research component and encourage faculty to integrate these elements when possible*. A curriculum audit can reveal additional opportunities for hands-on learning.
- *Faculty may benefit from professional development focused on implementing graduate-course-based chemistry education research experiences*. While these models are inspired by undergraduate research-based pedagogy, inquiry-driven projects,^38^ iterative experimentation, and collaborative investigation can be tailored for master’s-level chemistry courses.
- *Emphasize the importance of research experience during the program*. The importance of research experience in finding a job after graduation should be emphasized so that students maximize the research opportunities available to them. Mentoring and advising can be used to encourage students to gain as much research experience as possible before graduation.

### 5.2 Theme #2: The Importance of Professional Skills

Students and alumni interviewed felt communication skills are important in the master’s program and for future workplaces. “I think communicating clearly, openly, and effectively is probably equally important as what you actually do in the lab,” said a Montclair M.S. student. “You want people to understand what you’re doing and give you good feedback.” Students valued opportunities to practice public speaking through the student research symposium and networking events, with some suggesting making participation mandatory. “I know a lot of students struggle with public speaking, but it’s such an important skill to have as you enter the workforce,” commented a Montclair M.S. student. “I think the student research symposium that we do is a great way to get more comfortable with that, but it’s not always a requirement to present your research.” Some students noted that team projects helped them develop communication and collaboration skills.

In a previously published survey, chemical industry professionals ranked students’ skills in the teamwork and self-efficacy categories significantly higher compared to chemistry faculty.^17^ Faculty included oral presentations in chemistry master’s courses, but this varied “I don’t ask students to do anything like that currently,” noted one faculty member. “I mean they get a chance to write stuff and see if I can understand what they’re talking about for the exams. But other than that, there isn’t anything specific for communication skills.” Faculty also shared examples of how they foster writing skills. “In the course that I taught last semester, I required students to write short reports twice during the course of the semester,” a faculty member said. “And then as a final project, I required them to present information verbally. And I point out to them that the purpose of this is to develop those written and oral communication skills.”

Industry representatives emphasized professional skills for training chemistry master’s students. “All the scientists we hire are brilliant, have research skills,” said an industry representative. “It’s your soft skills that distinguish you. But if you want to get your foot in the door, it’s going to be your research skills and how you present a presentation. So presentation skills are very, very important in how you conduct yourself at an interview.” Eight of nine industry representatives interviewed highlighted the importance of communication skills, five highlighted collaboration skills, and four highlighted problem-solving skills. They emphasized the importance of these skills for team-based work required in industry, as well as the importance of these skills for career advancement. “When we interview, I look a lot for soft skills,” reflected an industry representative. “I look for learning agility because I don’t think whatever you spend two years researching is what you’re going to do when you get here. I want to see the examples of where you problem-solve. I want you to be able to talk about them with me. I want to see that you can learn and you can learn differently, you have the resources to do that, and you’re not going to operate in a silo. We no longer sit in the back of a hood like they did in the ‘50s and work all by yourself.” These findings support integration of professional skill development and networking opportunities for students to engage in communities of practice, facilitating legitimate peripheral participation where students gradually transfer from novice to expert roles, developing a professional identity and career readiness.

Based on student and industry input, the department expanded structured professional development. Starting in 2025, all master’s students are encouraged to present at the annual student research symposium before graduation. Career panels with alumni and industry were expanded, leading to internship and job placements. Selected graduate courses now integrate team work, primary literature, data analysis using Excel, short presentations, and written reports to build communication skills.

*Lessons Learned*

- *Map the specific and consistent ways that master’s students practice communication and collaboration*. Ensure exposure to a minimum level of professional skills training for all students before graduation.
- *Cultivate university- industry partnerships with local industry to provide mentorship, internship and networking opportunities, and resource sharing*. Adjunct faculty from industry can teach cutting-edge technologies.

### 5.3 Theme #3: Credit Load and Flexible Schedules

Students generally appreciated recent M.S. program changes at Montclair State, such as reduced credit requirements and greater emphasis on lab work and research. Concerns included limited elective offerings, limited research lab positions, the number of credit hours awarded for research not matching the actual effort/time required, and access to funding. “I definitely think having some more choices for electives would be good,” a Montclair M.S. student noted. “We have a decent amount of them that are listed, but nine times out of 10, they’re not even being offered that semester.” Core courses run regularly, but electives vary due to small program size.

Faculty also viewed recent changes positively, including changes to the program timeline, requiring fewer credits, and offering a more flexible schedule. They emphasized the different needs of full time students versus those working in industry. “There are some students who are full-time students, and then there are a few students who are working in industry,” said a faculty member. “I think we need to be a little bit more mindful about people who are currently not working and it’s a full-time experience, and their experience is a little bit different than people who are already out there in the workforce.”

Certificate or specialization programs were not a clear priority. Faculty highlighted the master’s programs as Ph.D. stepping stones, particularly for students from underrepresented groups.^39^ Industry representatives said certificates rarely affect hiring decisions with ne representative commenting that certificates are “never something that we kind of look at and say yes or no. We don’t really base a candidate off of it.” When interviewees thought a certificate would be helpful, there was no consensus on what it should be. Ideas included business operations, instrumentation, pharmaceutical biochemistry, communication, environmental safety, organic chemistry, and computational chemistry.

Reducing credit requirements can help working students participate in research, but departments should prioritize maintaining core disciplinary competencies, while identifying lower-impact, elective requirements that could be removed. Working students benefit from evening or weekend courses, but this may require faculty workload adjustments. We identified sufficient faculty who were willing to teach graduate courses in the evenings to cover the required courses. However, in a department where faculty do not prefer to teach in the evenings, flexible scheduling may benefit from rotating evening teaching assignments, offering hybrid formats, or sharing teaching across departments in interdisciplinary courses. Questions that a department might consider include:

1. Which requirements are essential to retain while reducing credit hours?
2. How can part-time working students access research and professional development typically scheduled during working hours?
3. How can faculty workload policies support evening or hybrid courses without undue burden?

*Lessons Learned*

- *Balance the needs of full- and part-time students*. Working students may miss opportunities if they only participate in the evenings. Ensuring access to professional and hands-on research opportunities for all students should be a priority.
- *Weigh the value of certificates carefully*. Certificates may not enhance employability as much as strengthening research and professional skills.

### 5.4 Theme #4: Program Size

The size (enrollment) of Chemistry and Pharmaceutical Biochemistry master’s programs can be a key issue impacting the programs’ sustainability. A challenge at Montclair State is the wider university’s emphasis on high enrollment for a program to be viable. Within a small program like Montclair’s Chemistry M.S. program, specialized electives (e.g., relating to new technologies) become even more difficult to justify, given the small number of students that enroll in these courses. However, some faculty and deans said these specialized classes are important to the relevance and development of the program.

Offering more flexible schedules and course formats, as discussed in Theme #3, can also help keep small programs sustainable. Evening and online/hybrid courses make the program possible for more working students, which can increase enrollment. More students can mean larger classes, which helps justify keeping the program. Sharing classes with other departments or letting advanced undergraduates take graduate courses can also help increase enrollments. These steps can make the program more sustainable while still providing students strong training.

*Lessons Learned*

- *Use course substitutions*. Many biology and environmental science courses are chemistry-focused. Substitutions may require a program modification to increase flexibility. Posting a clear list of approved substitutions facilitates advising and improves transparency.
- *Leverage interdisciplinary synergies*. Cross-listing courses with other small programs can grow class sizes, sustain specialized topics, and improve students’ interdisciplinary collaboration skills. Programs should keep a clear list of approved cross-listed courses to avoid confusion.
- *Emphasize outcomes*. Chemistry and Pharmaceutical Biochemistry M.S. graduates at Montclair State move into high-paying jobs and leadership roles. Sharing career outcomes through a program Web sites and/or alumni panels shows administrators the program’s value.
- *Strengthen industry partnerships*. Partnerships with industry can showcase the value of the program and generate allies who can speak in support of the program. Internship opportunities can be an effective way to build industry relationships.
- *Explore undergraduate enrollment options*. Allowing advanced undergraduates to take graduate courses can bolster enrollment and address program viability.

## 6 Conclusions and Perspectives

This article discussed adapting Master’s-level chemistry education for future student and industry needs, based on a review of the Chemistry M.S. and Pharmaceutical Biochemistry M.S. programs at Montclair State University, a Hispanic Serving Institution located in the New Jersey pharmaceutical hub. Four key themes emerged: (1) the critical role of hands-on research and laboratory project experiences, (2) the need for structured professional skills development integrated into coursework, (3) the importance of flexible course schedules and credit requirements, and (4) the challenges of sustaining small programs.

The insights from this study present actionable recommendations for institutions seeking to modernize their Chemistry M.S. programs while addressing the unique needs of today’s diverse graduate student population. It is important to note that program evaluation differs from a formal research study in that its focus is on utility and improvement, rather than generalizable knowledge. The evaluation results detailed in this article show how Montclair has used evaluation findings to improve its offerings, and these “lessons learned” likely have utility for other institutions, but we do not attempt to claim that these insights will apply to all institutions.

Several program modifications have been implemented at Montclair as a result of the evaluation findings, including laboratory modules in core courses, integration of professional skills training to course work, and credit adjustments to allow part-time students to participate in research. Evaluation of these changes is planned, and findings will be reported in the future.

This work highlights the need for further research into how varying types of experiential learning experiences and structured professional skill development impact master’s-level chemistry students’ cognitive development and professional identity formation. Future studies should investigate the long-term effects of course-based laboratory projects and course-based professional skill development on career advancement, professional identity formation, and workforce alignment.

Seen through the lens of experiential learning theory, it is recommended that master’s-level chemistry programs incorporate structured hands-on research experiences, both as independent research options and as class-based lab projects, to deepen conceptual understanding and enhance problem solving skills. Additionally, providing students communities of practice via networking opportunities and integrated professional skill development will enhance their professional identity development and career readiness.

## Supplementary Material



## Notes

Interview protocols (Faculty and Staff Focus Group Guide, Chemistry M.S. Student Focus Group Guide, and Industry Interview Guide) (PDF)
