# Supplementary material for: Adapting Master’s-Level Chemistry Education in the United States: Insights from Industry, Academia, and the Literature
Source: J Chem Educ. 2025 Dec 12;103(1):195–202. doi: 10.1021/acs.jchemed.5c00418 (PMC12805566; doi:10.1021/acs.jchemed.5c00418)
Supplement: Supplementary file 1 [file ed5c00418_si_001.pdf]

Supporting Information

Adapting Master's-level Chemistry Education in the United States: Insights from Industry, Academia, and the Literature

Brian Johnson[1], Amanda Lindell[1], and Nina M. Goodey*[,2]

[1]Inform Evaluation & Research, Amherst, Massachusetts, USA.

[2]Department of Chemistry and Biochemistry, Montclair State University, Montclair, New Jersey, USA.

*Corresponding author: Nina M. Goodey, email: goodeyn@montclair.edu, ph: 973-666-1368

Supporting information below contains the following interview protocols: Faculty and Staff Focus Group Guide, Chemistry M.S. Student Focus Group Guide, and Industry Interview Guide

Montclair State University S-STEM
Faculty and Staff Focus Group Guide

Introductory Remarks

- *THANKS for your time!*
- *We are conducting this focus group to get your feedback on changes to the structure of the Chemistry M.S. program.*
- *This will take about 45 minutes.*
- *We're interested in your open and honest feedback and perspective because that is where some of the most useful learning comes from. We may use quotes in our reports, but we will only do so in an anonymous way that cannot be connected to specific individuals. However, because this is a focus group, we cannot guarantee confidentiality.*
- *Your participation is entirely voluntary. You can stop at any time (including now).*
- *You are welcome to skip any question, but we encourage you to answer as many questions as you can. Also please leave space for others if you feel like you have been talking a lot.*
- *I'll be recording the focus group. Afterward, the recording will be transcribed and then the recording will be destroyed. Do I have your permission to record the focus group?*
- *Questions or concerns?*

Questions

Changes to the Chemistry M.S. program in recent years have included: reducing the required number of credits for graduation, the opportunity to apply more research credits to degree requirements, and the ability to complete the program in 15 months. How do you feel about those changes?

- In what ways have the changes been successful?
- In what ways have the changes enhanced the student experience?
- Have these changes created any new challenges?

Not including their research or thesis component, the average Chemistry M.S. student at MSU completes 7 classes (21 credits) to fulfill their degree. Approximately what percentage of the time spent in those classes do you think should be spent as lab time vs. lecture?

- Why did you give that answer?
- Under what circumstances would you recommend increasing lab time vs. lecture time?
- Should all Chemistry M.S. courses have a lab component?
- What are the barriers or challenges associated with increasing lab time?

Some of our recent data from students and industry suggest that these two groups value hands-on experience as a core part of an M.S. degree. Do you agree or disagree?

- Why or why not?
- What does this look like in your classes? What are the challenges to doing more hands-on work in your classes and the M.S. program as a whole?

- How can your department increase hands-on learning opportunities for M.S. students?

Our recent data also suggest that students and industry value effective communication skills, both written and verbal. How good of a job do you think the current M.S. program does at fostering the development of communication skills?

- What changes do you recommend to strengthen these skills in students?

What else do students need to be successful in chemistry careers that they aren’t currently getting from the M.S. program, if anything?

Are there any other changes you think should be made to the Chemistry M.S. program?

- Why do you recommend those changes?

Is there anything else you would like to tell me about the Chemistry M.S. program at Montclair?

Montclair State University S-STEM
Chemistry M.S. Student Focus Group Guide

Introductory Remarks

- *THANKS for your time!*
- *We are conducting this focus group to get your feedback on the Chemistry M.S. program.*
- *This will take about 45 minutes.*
- *We're interested in your open and honest feedback and perspective because that is where some of the most useful learning comes from. We may use quotes in our reports, but we will only do so in an anonymous way that cannot be connected to specific individuals. However, because this is a focus group, we cannot guarantee confidentiality.*
- *Your participation is entirely voluntary. You can stop at any time (including now).*
- *You are welcome to skip any question, but we encourage you to answer as many questions as you can. Also please leave space for others if you feel like you have been talking a lot.*
- *I'll be recording the focus group. Afterward, the recording will be transcribed and then the recording will be destroyed. Do I have your permission to record the focus group?*
- *Questions or concerns?*

Questions

Changes to the Chemistry M.S. program in recent years have included: reducing the required number of credits for graduation, the opportunity to apply more research credits to degree requirements, and the ability to complete the program in 15 months. How do you feel about those changes?

- How, if at all, have these changes impacted your student experience?
- Have you faced any barriers to participating in the M.S. program?

Not including their research or thesis component, the average Chemistry M.S. student at MSU completes 7 classes (21 credits) to fulfill their degree. Approximately what percentage of the time spent in those classes do you think should be spent as lab time vs. lecture?

- Why did you give that answer?
- Should all Chemistry M.S. courses have a lab component?

Tell me about the thesis component within the M.S. program. If you are pursuing a thesis, why? If you are not pursuing a thesis, why not? Is there anything the program could do to enable you to pursue a thesis?

How important do you think hands-on experience is as a core part of a Chemistry M.S. degree?

- How valuable would an internship experience be if it were offered through Montclair? What might be the challenges for you to participate in an internship?

How important do you think the development of communications skills is as a core part of a Chemistry M.S. degree?

Are there any other professional skills you would like to develop through the Chemistry M.S. program? Why?

What is the biggest challenge or obstacle you have faced participating in this program?

- What can the program to do help alleviate or address this challenge?

What do you think needs to change in the Chemistry MS program in the future?

- Why do you recommend those changes?

Is there anything else you would like to tell me about the Chemistry M.S. program at Montclair?

Montclair State University S-STEM
Industry Interview Guide

Introductory Remarks

- *THANKS for your time!*
- *We are conducting this interview to help Montclair make changes to its Chemistry M.S. program.*
- *This will take about 15-20 minutes.*
- *We're interested in your open and honest feedback and perspective because that is where some of the most useful learning comes from. We may use quotes in our reports and articles, but we will only do so in an anonymous way that cannot be connected to specific individuals.*
- *Your participation is entirely voluntary. You can stop at any time (including now).*
- *You are welcome to skip any question, but we encourage you to answer as many questions as you can.*
- *I'll be recording the interview. Afterward, the recording will be transcribed and then the recording will be destroyed. Do I have your permission to record the interview?*
- *Questions or concerns?*

Questions

What skills or competencies do you think should be prioritized in a Chemistry M.S. or Pharmaceutical Biochemistry M.S. program?

- Why did you give that answer?

When hiring a position suitable for a recent Chemistry M.S. or Pharmaceutical Biochemistry M.S. graduate, what would give an applicant a competitive edge at your company?

What role do you think a Chemistry M.S. or Pharmaceutical Biochemistry M.S. program should play in fostering non-technical skills such as communication, collaboration and problem-solving?

- How do you think those skills can be effectively taught in an M.S. program?

If any, what specializations or certificates would be appealing to you when hiring a Chemistry M.S. or Pharmaceutical Biochemistry M.S. graduate?

Do you have any other recommendations for Montclair as it seeks to best position its Chemistry M.S. or Pharmaceutical Biochemistry M.S. program to meet the future needs of students and industry?
